# Choice Matters More with Others: Choosing to be with Other People is More Consequential to Well-Being than Choosing to be Alone

**DOI:** 10.1007/s10902-022-00506-5

**Published:** 2022-03-02

**Authors:** Liad Uziel, Tomer Schmidt-Barad

**Affiliations:** 1 Department of Psychology, Bar-Ilan University, 5290002 Ramat-Gan, Israel; 2 Peres Academic Center, Rehovot, Israel

**Keywords:** Social presence, Aloneness, Solitude, Choice, Subjective well-being, Sense of meaning

## Abstract

Stable social relationships are conducive to well-being. However, similar effects are not reported consistently for daily social interactions in affecting episodic (experiential) subjective well-being (ESWB). The present investigation suggests that the choice of being in a social context plays an important moderating role, such that social interactions increase ESWB only if taken place by one's choice. Moreover, it is argued that choice matters more in a social context than in an alone context because experiences with others are amplified. These ideas were tested and supported in two studies: An experiment that manipulated social context and choice status, and a 10-day experience-sampling study, which explored these variables in real-life settings. Results showed that being with others by one’s choice had the strongest positive association with ESWB, sense of meaning, and control, whereas being with others not by one’s choice—the strongest negative association with ESWB. Effects of being alone on ESWB also varied by choice status, but to a lesser extent. The findings offer theoretical and practical insights into the effects of the social environment on well-being.

## Introduction

Modern days present individuals with ever-growing possibilities for managing their social lives. Developments in computing, communication, and transportation, introduce novel channels for social interactions, alongside increased opportunities for spending time alone productively (e.g., Verduyn et al., 2021). The present investigation is focused on these basic social conditions (alone/with others), on the choice of being in these contexts, and on their joint (interactive) effect on well-being.

The tendency to form and maintain social bonds is considered a fundamental need (Kenrick et al., 2010; Maslow, 1943), expressed in humans from infancy and across the lifespan (Baumeister & Leary, 1995; Bowlby, 1973; House et al., 1988; Stevens & Fiske, 1995). Its fulfillment has positive effects on well-being (e.g., Deci & Ryan, 2000; Diener & Seligman, 2002; Myers, 2000; Reis et al., 2000), whereas dearth of social relations carries dire consequences such as depression and poor health (Hawkley & Cacioppo, 2010; House et al., 1988).

Notwithstanding, much of the evidence associating social relations with subjective well-being (SWB) is based on general and static (i.e., stable status) estimations of the variables, such as in the association between one's marital status and general satisfaction with life (Myers, 2000). These findings are highly informative and of much value, but they do not necessarily reflect the effects embedded in the dynamics of everyday *social interactions* on experiential (i.e., episodic) SWB (Hudson et al., 2020). That is, knowing that solid social bonds contribute to general satisfaction with life does not inform about the association of momentary social interactions with momentary SWB, because these variables carry different meanings at the two levels of analysis (global vs. specific). *Experiential subjective well-being* (ESWB) reflects the mood and emotions that people experience in a given context (Hudson et al., 2020). Research indicates that it is only loosely related to measures of global well-being (Kim-Prieto et al., 2005). Moreover, static social relations are usually based on having prolonged, frequent contacts with a few highly liked individuals, whereas everyday social contacts are diverse, unpredictable, and often brief (Del Valle et al., 2007).

Using diary, day reconstruction, and experience sampling methods, research has started to explore the association of daily social interactions with ESWB. Nezlek et al. (2002) reported that ESWB gained by merely being more socially active, and more so by having rewarding interactions. A similar conclusion was reached by Larson et al. (1985) involving a population of retired adults. More recently, Sandstrom and Dunn (2014) reported greater happiness and well-being on days with many social interactions, and Wilt and Revelle (2019) associated social interactions with energic arousal. Sun et al. (2020) reached a similar conclusion, connecting the sheer quantity of social interactions with momentary well-being (retrieved from self-reports and proxy reports).

However, other studies have indicated that social interactions do not always bring about greater ESWB. Hudson et al. (2020) emphasized that different interaction contexts yield different experiences (see also Lucas & Dyrenforth, 2006). Similarly, in Kahneman et al. (2004), social interactions were both among the most likable daily activities (e.g., intimate relations), but also among the least likable activities (e.g., caring for one's children), indicating that social contacts carry a mixed bag of effects. Uziel et al. (2020) found no direct relation between momentary social context (alone/’with others’) and reported happiness, whereas Rook (1984) highlighted potential destructive effects of negative daily contacts on ESWB (see also Rafaeli et al., 2008). Taken together, research focusing on real-life experiences shows that social interactions may be conducive to ESWB, but the experience is subjected to substantial variations that need to be better understood.

Does time alone contribute systematically to well-being? Recent years have seen a surge in research on solitude, and with it in findings associating solitude to well-being (Coplan & Bowker, 2014). Research often reports that solitude carries detrimental effects on well-being. For example, Larson (1990) reported that the experience of solitude in daily life is one of loneliness and passivity and that spending extended time alone is a marker of poor adjustment. Matias et al. (2011) reported increased levels of stress hormones when alone, whereas Teppers et al. (2013) found that traits reflecting emotional stability were associated with an aversion of aloneness (see also Lay et al., 2018; Uziel, 2016). Behaviorally, Wilson et al. (2014) reported that people find being alone extremely aversive and would rather keep themselves occupied in self-harming (yet stimulating) activities. Notwithstanding, research also documents desired effects for solitude, stressing its contribution to well-being. Time alone arises in these studies as an opportunity to relax, regulate emotions, and reflect on one's life, thus enhancing and consolidating one’s selfhood (Coplan & Bowker, 2014; Long & Averill, 2003; Nguyen et al., 2018; Pauly et al., 2018; Uziel, 2021). The factors that determine the nature of the alone experience are the focus of emerging yet still indecisive research (Coplan et al., 2019; Lay et al., 2018; Uziel et al., 2020).

Real-life data that compares the alone context with being with others indicates that, on average, the latter is more conducive to ESWB. For example, in Kahneman et al. (2004), being alone was associated with lower positive affect (but not higher negative affect) compared with social activities involving other people. Similar results were reported in Srivastava (2008) using an experience-sampling method, in Csikszentmihalyi and Hunter (2014) among primary school students, and by Choi et al. (2017) and Hudson et al. (2020) on adult samples. Yet, given the variability of experiences in both conditions, this question needs to be further explored by addressing potential moderators, which may convey a more complete picture of the dynamics taking place.

As potential moderators of the effects of daily social interactions, research has frequently explored objective parameters of the setting (e.g., number of other people, partner identity, weekday). Notwithstanding, these factors oftentimes do not convey systematic variations in experience. For example, as emerging from the findings of Kahneman et al. (2004) and Epley and Schroeder (2014), the closeness of one's interaction partner (e.g., your child vs. a stranger in the subway) sometimes carries unexpected effects on ESWB and is therefore not a dependable indicator.

A factor that has not attracted much attention but holds a potential to play an important role as a moderator is whether the setting is elected or imposed. That is, whether the person is in social interaction (or alone) by personal choice or because of external circumstances. Research involving real-life data has addressed the question of choice with only a handful of studies, whereas lab-based experimental data is almost entirely focused on imposed social settings. Tentative evidence for the role of elected social contacts on ESWB comes from an experience sampling study by O'Connor and Rosenblood (1996). Their research found that in 66% of the sampling points, participants were in a social condition (alone/with others) of their preference (and thus, in 34% they were in a non-elected context). Being in an elected social condition predicted the likelihood of remaining in this setting (thus implying better ESWB). Similar findings were reported in Hall (2017) using a larger and more diverse sample (here, 53% of participants’ reports indicated being in a non-elected setting). In one of the only direct explorations of the effect of elected social contexts on ESWB, Hall and Merolla (2020) reported that on days in which participants felt that they had a choice about how to spend their social time they reported higher life satisfaction. Similarly, Nezlek (2001) reported that psychological adjustment was higher on days when participants' socializing plans were realized. Taken together, initial evidence indicates that choice is a substantial moderator of the effect of social interactions on ESWB, alongside findings suggesting that it plays a key role in the alone experience as well (Coplan et al., 2019; Lay et al., 2018). However, additional research is needed, focusing on people’s experiences during (i.e., beyond their general retrospection about) their social interactions and during their time alone—goals that the present investigation aims to achieve.

Moreover, existing research does not address the question of whether choice carries *a different impact* on well-being when one chooses to be alone versus when one chooses to be with others—a question at the core of the present investigation. Reason has it that volition plays a stronger effect in the 'with others' context. Research indicates that relative to alone experiences, social interactions are highly arousing (Bond & Titus, 1983; Zajonc, 1965), and mentally demanding (Baron, 1986; Lieberman & Rosenthal, 2001; Uziel & Baumeister, 2012). Self-presentational concerns cause events happening with others to carry greater weight (Baumeister, 1982). As a result, responses to social presence are known to be either highly facilitative or highly inhibitive compared to alone settings (Uziel, 2007). More so, recent experimental findings have indicated that events taking place during social interactions are amplified and perceived to be stronger, for better or worse (Boothby et al., 2014; Steinmetz et al., 2016). These characteristics set the stage for more extreme reactions to similar processes when they occur in a 'with others' context than in an alone context. Taken together, choice is likely to affect well-being in both settings, alone and 'with others', but its effect will be more consequential under the 'with others' condition.

### The Present Research

We report two studies that used different methods to test our prediction that choice (specifically, perceived choice) has a more substantial impact on ESWB in a ‘with others’ context than in an alone context. Study 1 was an experiment that sought to control for exogenous variables. Study 2, our main study, applied an experience-sampling method to test our prediction in real-life dynamics.

### Statistical Power and Open Science Declaration

Sample size considerations and statistical power calculations are reported at the introduction of each study. All materials^1^, data, and code are posted at the Open Science Framework: https://osf.io/tukwe/

## Study 1

### Overview

In Study 1 we sought to test the effect of choice to be alone/'with others' on ESWB in a controlled setting (complementing the real-life setting from Study 2). We specifically sought to control for the physical setting, because people’s (chosen vs. not) social experiences are often attached to specific environments (a factor we could not control in real-life settings). To this end, we asked participants to report about their ESWB while instructing them to consider a fixed physical environment (“at home”). If, as outlined in the Introduction, choice is more consequential to well-being 'with others' (vs. alone) because of the psychological impact of others’ company, its differential effect should show even when controlling for the physical environment. To further substantiate this idea, we also asked the participants to consider another environment (“at work”), which often introduces a different probability of being alone (and with others) by choice (or not) than the home setting. We expected to repeat the interaction of choice by social context from the “at home” setting in the “at work” setting.

A-priory power analysis (using G*power; Faul et al., 2009) advised that detecting mean difference of *d* = 0.50 with 80% power in the (2-by-2) within-subject design of the present study requires a sample size of *N* = 34. Given that we were mainly interested in the interaction of choice and social setting, we followed with another power analysis using simulation-based software (Lakens & Caldwell, 2021). This analysis advised that 70 participants are required to detect a small-medium effect (η^2^_p_ = 0.11) with 80% power. Our final sample has exceeded this.

## Method

### Participants and Procedure

Participants (*N* = 85) were recruited via the Prolific Academic platform (https://www.prolific.co) to a study on 'life experiences and subjective well-being**'.** The sample comprised of 52 Females, 33 Males, *M*_age_ = 32.85 (*SD*_age_ = 12.50), residents of the United Kingdom (*n* = 83) and the USA (*n* = 2). All respondents were included in the analysis.

The study had a within-subject design. Thus, all participants experienced the same procedure (albeit the presentation of stimuli varied randomly). On entering the study, participants signed a consent form and proceeded to the main part, which comprised of two questionnaires asking them to rate their ESWB. After completing their ratings, participants were thanked and compensated.

### Materials

Two 4-item questionnaires were presented in random order.

#### ESWB ‘at Home’

Participants were instructed to think of themselves while *at home*. Next, they read 4 scenarios (presented in random order) depicting social context (“alone”/ “with other people”) and choice status for being in this context (“by your choice”/ “because of external circumstances”). For example: “[At home] alone, by your choice” or “[At home] with other people, because of external circumstance (that is, you would rather not be with those people)”. For each of the 4 scenarios, participants rated their level of satisfaction on a 1 = *not satisfied* to 7 = *very satisfied* scale.

#### ESWB ‘at Work’

The same 4 scenarios from the ‘at home’ setting, this time instructing participants to think of themselves while at work.

## Results and Discussion

Table 1 presents the Means and *SDs* of the satisfaction ratings across the different settings. As seen in Table 1, for both 'at home' and 'at work', the most satisfying setting was 'with others' by one's choice, and the least satisfying—'with others' not by one's choice. Levels of satisfaction alone were in between the 'with others' conditions.

**Table 1:** Satisfaction (M, SD) from being alone/'with others' by choice (vs. Not) in two physical settings (Study 1)

Context	At home	At work
With others, by my choice	5.95 (1.06)	5.52 (1.28)
With others, not by my choice	3.05 (1.71)	3.25 (1.60)
Alone, by my choice	5.58 (1.55)	5.36 (1.48)
Alone, not by my choice	3.28 (1.60)	3.78 (1.42)

*N* = 85

To formally test our prediction about the differential effect of choice in the 'with others' context as compared with the alone context, we conducted a 2 (alone/'with others') by 2 (my choice/not my choice) repeated measures ANOVA. Focusing first on the 'at home' setting, the analysis revealed a main-effect for choice, *F*(1, 84) = 207.68, *p* < 0.001, η^2^_p_ = 0.712, with satisfaction higher in the 'my choice' condition (*M* = 5.76; *SD* = 0.98) compared with 'not by my choice' (*M* = 3.16; *SD* = 1.31). No effect was found for social context, *F*(1, 84) = 0.18, *p* = 0.68, η^2^_p_ = 0.002, addressing the difference between the alone context (*M* = 4.42; *SD* = 1.19) and the 'with others' context (*M* = 4.50; *SD* = 1.04).

Importantly, the analysis revealed a significant interaction between social context and choice status, *F*(1, 84) = 6.48, *p* = 0.013, η^2^_p_ = 0.072, reflecting a larger discrepancy between the two choice conditions in the 'with others' context (*M* = 2.90, *SD* = 1.93) than in the alone context (*M* = 2.29, *SD* = 2.06). That is, results from the 'at home' context indicated that choice makes a larger difference in a 'with others' context than an alone context, even when the physical setting is controlled.

We followed with another repeated measures ANOVA, focusing on the 'at work' setting. The analysis revealed a main-effect for choice, *F*(1, 84) = 100.65, *p* < 0.001, η^2^_p_ = 0.545, with satisfaction higher for the 'my choice' condition (*M* = 5.44; *SD* = 1.08) compared with 'not by my choice' (*M* = 3.51; *SD* = 1.24). No effect was found for social context, *F*(1, 84) = 1.84, *p* = 0.18, η^2^_p_ = 0.02, addressing the difference between the alone context (*M* = 4.57; *SD* = 0.97) and the 'with others' context (*M* = 4.38; *SD* = 1.01).

Importantly, the analysis yielded a significant interaction between social context and choice status, *F*(1, 84) = 7.12, *p* = 0.009, η^2^_p_ = 0.078, reflecting a larger discrepancy between the two choice conditions in the 'with others' context (*M* = 2.27, *SD* = 2.09) than in the alone context (*M* = 1.58, *SD* = 2.16). That is, results 'at work' replicated the findings from the 'at home' setting in terms of the greater impact of choice on ESWB 'with others' than alone.^2^

Taken together, results based on two different physical settings indicate that choice matters more in affecting ESWB in a 'with others' context than in an alone context. These findings indicate that the amplifying impact associated with choice in a 'with others' context reflects the psychological experience and not variation (or lack of) in physical settings. That is, people appear to experience being with others (and implications of realities unfolding when with others) as more consequential to their well-being. In Study 2, we sought to test these ideas over an extended period, across many events, and by looking at a wider range of expressions of ESWB.

## Study 2

### Overview

In our main study, we utilized an experience sampling method. We adopted this approach to maximize the ecological validity of the findings, seeking to address real-life variations in chosen (vs. not) social contexts by sampling within and across days. To this end, participants reported 3 times a day for 10 days (5 days a week for two weeks) about their social context and their choice of being in this context. They then reported about their ESWB, which included items about satisfaction with life and positive and negative affect. Participants also reported their *sense of meaning* in their current activities. To the extent that actions are magnified in social contexts (Steinmetz et al., 2016), being with others should be associated with a greater sense of meaning. In addition, we asked participants whether they currently feel *control over their life* expecting it to be mainly affected by whether they are currently in a situation of their choice. Like in Study 1, our focal interest was on the interaction between social context and choice, expecting choice to carry a more substantial effect on ESWB in the 'with others' context than alone.

Building on benchmarks retrieved from a Monte-Carlo simulation (Arend & Schäfer, 2019), we reasoned that with 150 participants, each providing 30 ratings, we can achieve 80% power for detecting small effects (*r* ≥ 0.09). Our final sample (*N* = 155) is consistent with that and is also in line with current norms in studies utilizing a similar design (Hudson et al., 2020; Lay et al., 2018).

## Method

### Participants and Procedure

Participants in this study (*N* = 155; Females = 125; Males = 30; *M*_age_ = 23.92, *SD*_age_ = 3.12) were Israeli students recruited through a university bulletin board to participate in a broad experience sampling research, aimed at learning about the implications of daily experiences on psychological states. As part of this broader study, personality questionnaires (not analyzed here), demographic data, and contact information were collected at a start-up session.

Episodic data were collected by prompting participants (with text messages to their phones) to report three times a day (morning, noon, and evening) for 10 consecutive days (weekends excluded). Episodic reports started by asking about the current social context (alone/with other people) and whether this social context was a result of one's choice or not. Participants then reported about their ESWB (positive and negative affect, satisfaction with life) and their sense of meaning and control.

### Materials

#### Social Condition

Participants reported with one question whether they are *currently* alone (coded 0) or with other people (coded 1). 'With others' was defined as being in the same space with other people, and/or actively communicating with other people. Alone was defined as being physically alone while not actively communicating with other people.

#### Choice

Participants reported with one question whether they are in their respective social condition (alone/with others) by their choice (coded 0) or not by their choice (i.e., because of external demands; coded 1).

#### Satisfaction with Life

Satisfaction with life was measured with 2 items (“I am satisfied with my life” and “The conditions of my life are excellent”) taken from the Satisfaction with Life Scale (Diener et al., 1985). Participants marked their agreement with each item (on a 1 = *strongly disagree* to 5 = *strongly agree* scale) based on their experiences *in the past few minutes* (α = 0.75).

#### Positive and Negative Feelings

Participants were presented with 4 items representing positive and negative feelings. Ratings were made with reference to *the past few minutes* along a 1 = *very little or not at all* to 5 = *very much* scale. Two items were used to measure positive feelings (“calm” and “excited; α = 0.51) and two items were used to measure negative feelings (“sad” and “tense”; α = 0.67).

#### Meaning

Participants were presented with 2 items (“My actions in the past few minutes have meaning” and “My actions in the past few minutes fill me with interest”) adapted from the Meaning in Life Questionnaire (Steger et al., 2006). Responses were anchored by 1 = *very little or not at all* to 5 = *extremely* scale (α = 0.79).

#### Control/Mastery

Participants reported along 2 items (“I can do just about anything I really set my mind to” and “What happens to me in the future mostly depends on me”) from the Pearlin Self-Mastery Scale (Pearlin & Schooler, 1978). Ratings addressed their feelings in *the past few minutes* on a 1 = *strongly disagree* to 5 = *strongly agree* scale (α = 0.73).

## Results

### Analysis Overview and Descriptive Statistics

Because of the clustered nature of the data (momentary reports nested within individual participants), results were analyzed by multilevel modeling with SPSS Mixed procedure, and jamovi's (The jamovi project, 2021) GAMLj module (Gallucci, 2019).

The 155 participants provided 4296 momentary reports, yielding a mean of 27.71 (*SD* = 5.98) reports per participant out of 30 potential reports, reflecting a high response rate (92.38%). Across all momentary reports, participants were alone 37.1% of the time and with other people 62.9% of the time. Participants were in a social condition of their choosing 64.3% of the time and in a social condition that is the result of external demand 35.7% of the time. When with others, participants were in 59.2% of the episodes by their choice (and in 40.8% not by their choice), whereas when alone, they were in 72.8% of the episodes by their choice (and in 27.2% not by their choice).

Table 2 presents Means, *SD*s, ICCs, and correlations of the study's variables. Note that for this analysis the Level-1 variables—momentary social condition, choice status, positive and negative affect, control mastery, sense of meaning, and satisfaction with life—were averaged within individuals and then across individuals. As seen in Table 2, people who, on average (i.e., across all episodes), were more often with other people also tended to be more often in non-chosen conditions, implying that non-chosen settings are somewhat more common with others than alone. Notwithstanding, people that, on average, spent more with others also reported a higher level of satisfaction with life. People who found themselves more often in non-chosen social settings reported a reduced sense of control, less meaning in their actions, and lower positive feelings (but not higher negative feelings). The analysis further indicated correlations between the expressions of ESWB (satisfaction with life, positive and negative feelings), which were also correlated with sense of meaning and control, as to be expected.

**Table 2:** Descriptive statistics of the main variables in Study 2

Variable	M(SD)	ICC	2	3	4	5	6	7
1. Social condition	0.63(0.17)	.073	.21^**^	.28^**^	.10	− .20^*^	.06	.23^**^
2. Choice status	0.36(0.19)	.118	**–**	− .11	− .23^**^	.01	− .20^*^	− .16^*^
3. Satisfaction with life	3.77(0.62)	.651		**–**	.30^***^	− .32^***^	.43^***^	.63^***^
4. Positive feelings	2.60 (0.56)	.310			**–**	− .39^***^	.55^***^	.42^***^
5. Negative feelings	1.75(0.54)	.348				—	− .19^*^	− .39^***^
6. Meaning	2.88(0.56)	.270					–	.37^***^
7. Control	3.66(0.53)	.492						–

*N* = 155

Social condition (0 = alone, 1 = with others); Choice status (0 = My choice, 1 = Not my choice)

* = *p* < .05, ** = *p* < .01, *** = *p* < .001

### Main Analysis

Our focal question involved the interactive effects of social conditions (alone/with others) and choice status (my choice/not my choice) in affecting ESWB. In all analyses, our predictors were social condition (Level-1 variable, dummy coded with 0 = alone and 1 = with others) and choice status condition (Level-1 variable, dummy coded with 0 = my choice and 1 = not my choice), followed by their interaction term (intercepts were allowed to vary randomly between individuals). We present the results by our dependent variables: Expressions of ESWB (satisfaction with life, positive and negative affect), sense of meaning, and control/mastery.

#### Satisfaction with Life

**Fig. 1:**
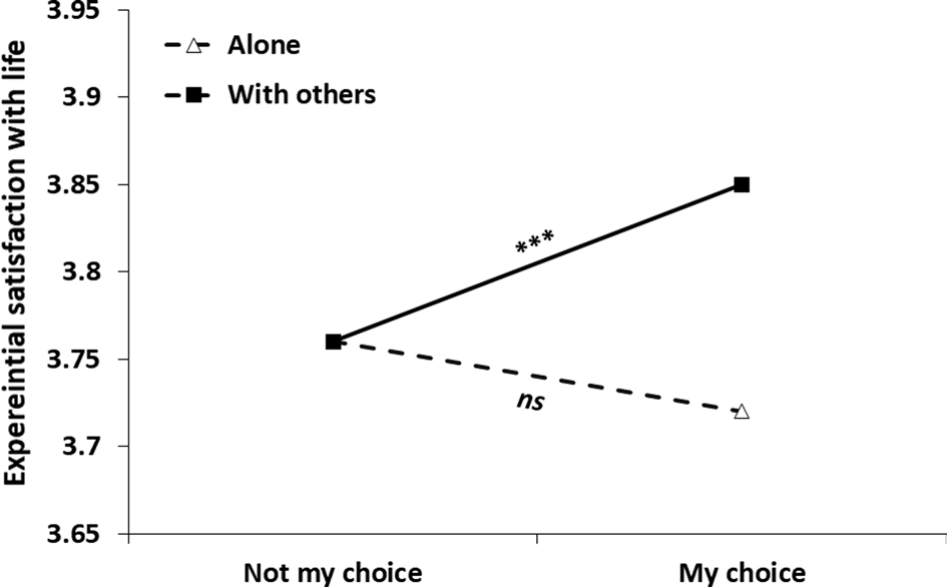
Experiential satisfaction with life as a function of social context and choice of being in this context. *Note*. *** = *p* < .001

Step 1 of the analysis (without an interaction term) yielded a main effect for social context, *b* = 0.09, *SE* = 0.01, 95% CI [0.06, 0.12], *t*(4151) = 5.919, *p* < 0.001, indicating that participants reported greater satisfaction in the company of others, compared to when they were alone. A main effect was also found for choice status, *b* = − 0.05, *SE* = 0.02, 95% CI [− 0.08, − 0.02], *t*(4158) =  − 3.275, *p* = 0.001, indicating greater satisfaction when the social context was a result of choice, compared with a result of external constraints. Table 3 presents Step 2 of the analysis, after adding the interaction term between social condition and choice. As seen in the table, the main effect of choice was qualified by the interaction, indicating that choice had a different effect in the two social contexts (see Fig. 1). Specifically, choice had a significant effect in the ‘with others’ social condition, *b* = − 0.09, *SE* = 0.02, 95% CI [− 0.12, − 0.05], *t*(4154) =  − 4.84, *p* < 0.001, but it made little difference alone, *b* = 0.03, *SE* = 0.03, 95% CI [− 0.02, 0.09], *t*(4156) = 1.25, *p* = 0.21. Put simply, choice was more consequential in affecting episodic satisfaction with life 'with others' than alone.

**Table 3:** Results of a multilevel modeling analysis for predicting satisfaction with life from momentary social condition, choice status and their interaction (Study 2)

Parameter	*B*	*SE*	*t*	*p*
Intercept	3.71	0.05	73.33	< .001
Social condition^a^	0.13	0.02	7.04	< .001
Choice status^b^	0.03	0.03	1.25	.211
Social condition*Choice status	− 0.12	0.03	− 3.81	< .001

*N* = 155

^a^ = Social condition (0 = alone, 1 = with others)

^b^ = Choice status (0 = my choice, 1 = not my choice)

#### Positive Feelings

**Fig. 2:**
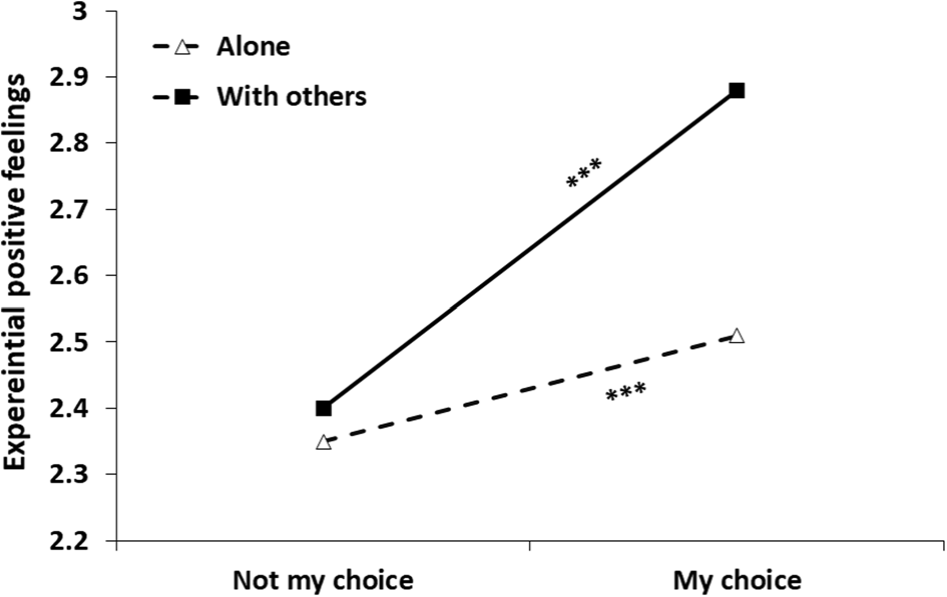
Experiential positive feelings as a function of social context and choice of being in this context. *Note*. *** = *p* < .001

Step 1 of the analysis yielded a main effect for social context, *b* = 0.26, *SE* = 0.03, 95% CI [0.21, 0.31], *t*(4198) = 10.33, *p* < 0.001, indicating more positive feelings in the company of others (vs. alone). A main effect was also found for choice status, *b* = -0.38, *SE* = 0.03, 95% CI [− 0.43, − 0.33], *t*(4223) = − 14.47, *p* < 0.001, indicating more positive feelings when the social context was by one's choice (vs. not). Table 4 presents Step 2 of the analysis, after adding the interaction term between social condition and choice. As seen in the table, the interaction was significant, indicating that choice had a different effect in the two social contexts (see Fig. 2). Specifically, choice had a significantly more substantial effect in the ‘with others’ social condition, *b* = − 0.48, *SE* = 0.03, 95% CI [− 0.54, − 0.42], *t*(4209) =  − 15.35, *p* < 0.001, than in the alone condition, *b* = − 0.16, *SE* = 0.05, 95% CI [− 0.25, − 0.07], *t*(4219) =  − 3.48, *p* < 0.001.

**Table 4:** Results of a multilevel modeling analysis for predicting positive feelings from momentary social condition, choice status and their interaction (Study 2)

Parameter	*B*	*SE*	*t*	*p*
Intercept	2.51	0.05	53.62	< .001
Social condition^a^	0.37	0.03	11.88	< .001
Choice status^b^	-0.16	0.05	− 3.48	< .001
Social condition*Choice status	-0.32	0.05	− 5.89	< .001

*N* = 155

^a^ = Social condition (0 = alone, 1 = with others)

^b^ = Choice status (0 = my choice, 1 = not my choice)

#### Negative Feelings

**Fig. 3:**
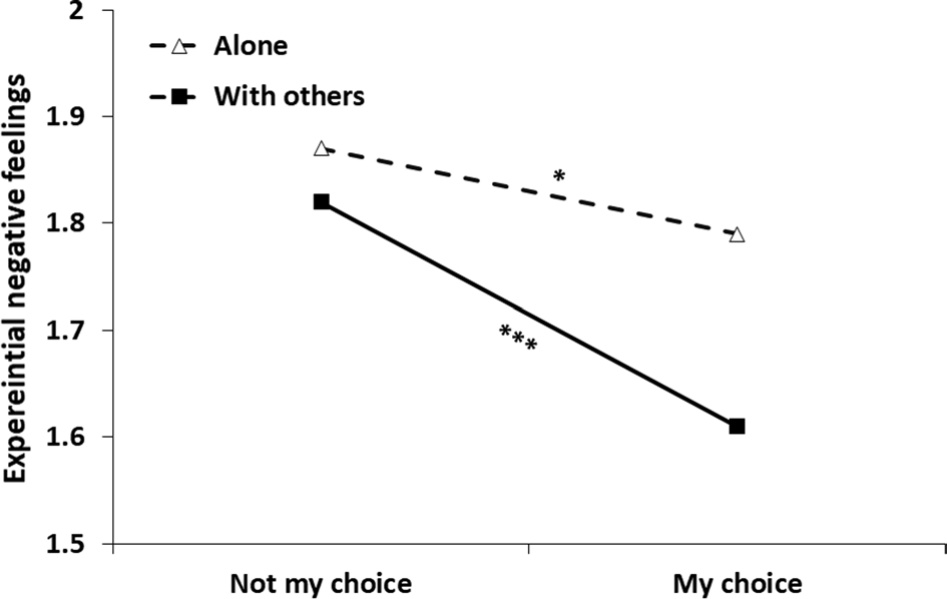
Experiential negative feelings as a function of social context and choice of being in this context. *Note*. *** = *p* < .001, * = *p* < .05

Step 1 of the analysis yielded a main effect for social context, *b* = − 0.13, *SE* = 0.02, 95% CI [− 0.18, − 0.09], *t*(4185) =  − 6.15, *p* < 0.001, indicating reduced negative feelings in the company of others (vs. alone). A main effect was also found for choice status, *b* = 0.17, *SE* = 0.02, 95% CI [0.12, 0.21], *t*(4208) = 7.31, *p* < 0.001, reflecting stronger negative feelings when the social context was not by one's choice. Table 5 presents Step 2 of the analysis, after adding the interaction term between social condition and choice. As seen in the table, the interaction was significant, indicating that choice had a different effect in the two social contexts (see Fig. 3). Specifically, choice had a significantly stronger effect in the ‘with others’ condition, *b* = 0.21, *SE* = 0.03, 95% CI [0.00, 0.16], *t*(4195) = 7.55, *p* < 0.001, than in the alone condition, *b* = 0.08, *SE* = 0.04, 95% CI [0.15, 0.26], *t*(4204) = 2.02, *p* = 0.04. Therefore, for negative feelings as well, choice was more consequential in affecting episodic feelings 'with others' than alone.

**Table 5:** Results of a multilevel modeling analysis for predicting negative feelings from momentary social condition, choice status and their interaction (Study 2)

Parameter	*B*	*SE*	*t*	*p*
Intercept	1.79	0.04	40.58	< .001
Social condition^a^	− 0.18	0.03	− 6.56	< .001
Choice status^b^	0.08	0.04	2.02	.044
Social condition*Choice status	0.13	0.05	2.64	.008

*N* = 155

^a^ = Social condition (0 = alone, 1 = with others)

^b^ = Choice status (0 = my choice, 1 = not my choice)

In sum, whether it was in reporting about their experiential satisfaction with life or their momentary affect ratings, choice had a notably stronger effect when people were with others as compared to when they were alone. In our next set of analyses, we have looked at additional variables, which shed more light on the overall experience.

#### Meaning

**Fig. 4:**
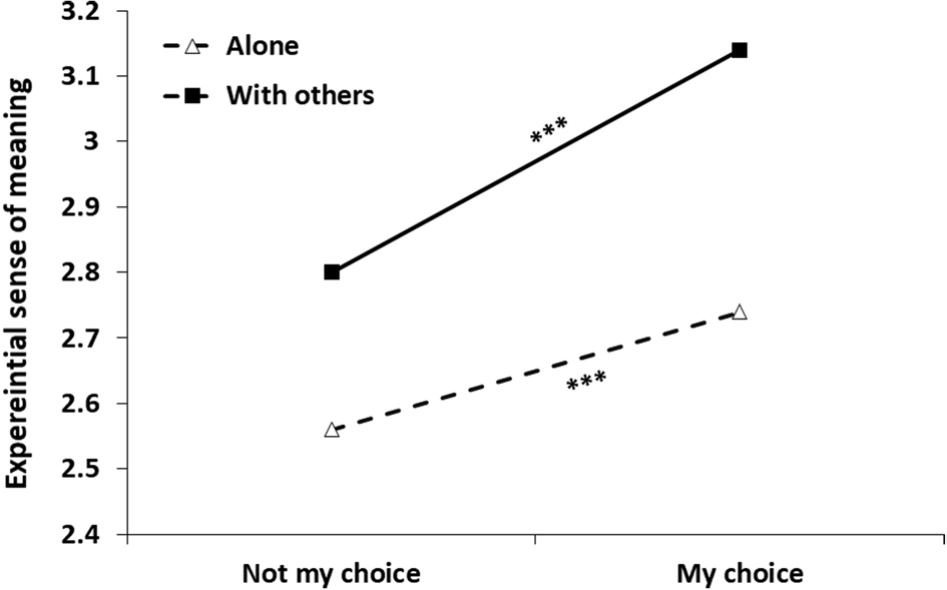
Experiential sense of meaning as a function of social context and choice of being in this context*. Note*. *** = *p* < .001

This set of analyses explored how meaningful participants experienced their time 'with others'/alone, when it was by their choice (vs. not). Step 1 of the analysis yielded a main effect for social context, *b* = 0.35, *SE* = 0.03, 95% CI [0.29, 0.40], *t*(4205) = 11.99, *p* < 0.001, indicating a stronger sense of meaning to activities occurring in the company of others (vs. alone). A main effect was also found for choice status, *b* = − 0.29, *SE* = 0.03, 95% CI [− 0.35, − 0.23], *t*(4232) =  − 9.72, *p* < 0.001, showing that actions taking place by one's choice are subjectively more meaningful. Table 6 presents Step 2 of the analysis, after adding the interaction term between social condition and choice. As seen in the table, the interaction was significant, indicating that choice had a different effect in the two social contexts (see Fig. 4). Specifically, choice had a significantly stronger effect in the ‘with others’ condition, *b* = − 0.34, *SE* = 0.04, 95% CI [− 0.41, − 0.27], *t*(4218) =  − 9.53, *p* < 0.001, than when alone, *b* = − 0.18, *SE* = 0.05, 95% CI [− 0.28, − 0.08], *t*(4228) = − 3.44, *p* < 0.001. Taken together, being with others was experienced as more meaningful especially when this was by one's choice.

**Table 6:** Results of a multilevel modeling analysis for predicting sense of meaning from momentary social condition, choice status and their interaction (Study 2)

Parameter	*B*	*SE*	*t*	*p*
Intercept	2.74	0.05	54.73	< .001
Social condition^a^	0.40	0.04	11.33	< .001
Choice status^b^	− 0.18	0.05	− 3.44	< .001
Social condition*Choice status	− 0.16	0.06	− 2.59	.010

*N* = 155

^a^ = Social condition (0 = alone, 1 = with others)

^b^ = Choice status (0 = my choice, 1 = not my choice)

#### Control

**Fig. 5:**
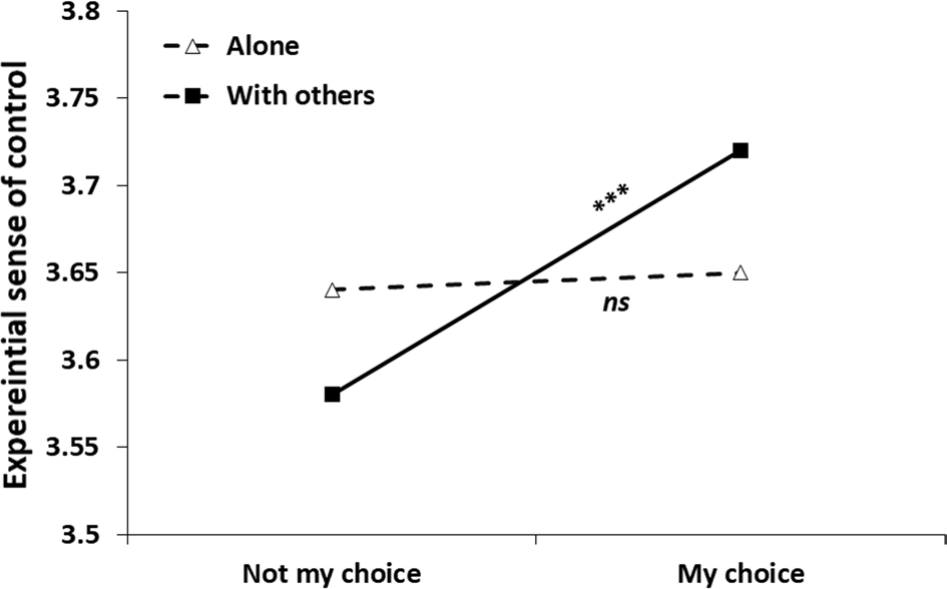
Experiential sense of control as a function of social context and choice of being in this context*. Note*. *** = *p* < .001

Our final set of analyses was focused on how much control participants experienced in the situations sampled. Step 1 of the analysis yielded no main effects for social context, *b* = 0.03, *SE* = 0.17, 95% CI [− 0.007, 0.061], *t*(4168) = 1.543, *p* = 0.123. A main effect showed for choice status, *b* = − 0.096, *SE* = 0.018, 95% CI [− 0.13, − 0.06], *t*(4182) =  − 5.31, *p* < 0.001, indicating that actions taking place in a social context of one's choice induced greater sense of control. Table 7 presents Step 2 of the analysis, after adding the interaction term between social condition and choice. As seen in the table, the main effect of choice was qualified by the interaction. Choice had a different effect in the two social contexts (see Fig. 5). Specifically, choice had a significant effect in the ‘with others’ condition, *b* = − 0.13, *SE* = 0.02, 95% CI [− 0.18, − 0.09], *t*(4174) = − 6.18, *p* < 0.001, but it made no difference in affecting sense of control alone, *b* = − 0.01, *SE* = 0.03, 95% CI [− 0.08, 0.05], *t*(4179) =  − 0.44, *p* = 0.658. That is, sense of control was experienced as stronger with others, mostly when it was by one's choice.

**Table 7:** Results of a multilevel modeling analysis for predicting sense of control from momentary social condition, choice status and their interaction (Study 2)

Parameter	*B*	*SE*	*t*	*p*
Intercept	3.35	0.04	82.16	< .001
Social condition^a^	0.07	0.02	3.07	.002
Choice status^b^	− 0.01	0.03	− 0.44	.658
Social condition*Choice status	− 0.12	0.04	− 3.17	.002

*N* = 155

^a^ = Social condition (0 = alone, 1 = with others)

^b^ = Choice status (0 = my choice, 1 = not my choice)

## Discussion

By studying participants’ experiences in their natural environment, this study affirmed our previous findings that ESWB is shaped by an interaction between the social context and choice of being in this context. Across the different expressions of ESWB, choice was more consequential 'with others' than alone, corroborating approaches that suggest that social contexts act to amplify and intensify experiences (e.g., Steinmetz et al., 2016).

The findings extended beyond ESWB, addressing some of the processes that could account for the observed differences in ESWB. Being with others by choice was also associated with an increase in sense of meaning and control. Our participants evaluated their activities and their level of agency more extremely during non-solitary experiences, and the choice of being in each social context moderated whether this would be for better or worse.

## General Discussion

Being alone and socializing are fundamental bricks in the human experience. The mere being in one state (vs. the other) carries important short-term (Kahneman et al., 2004; Uziel, 2007) and long-term (Bowlby, 1973; Winnicott, 1958) implications in a wide range of domains—affective, cognitive, motivational, and behavioral. Crucially, both conditions are conducive to well-being (Uziel, 2021). Seminal studies documented the immense importance of meeting social needs and establishing sound social bonds on healthy development and personal well-being (Baumeister & Leary, 1995), and emerging literature recognizes the benefits of solitary living (DePaulo & Morris, 2005).

In the present investigation, we sought to add to this literature in several respects. First, much of our knowledge on the effects of social bonds or solitary living is based on these conditions as stable ways of living (e.g., being married vs. being single). These are important aspects of our social lives, but the knowledge acquired only via a 'stable relations' lens does not capture the dynamics of our social lives as they unfold across the scenes that comprise our daily experiences (Nezlek et al., 2002). Second, research generally does not compare these social conditions (alone/'with others') but studies each condition separately. And, importantly, research has yet to fully account for the substantial variability in ESWB in these two settings. To address these issues we conducted two studies, an experiment and an experience-sampling study, which provided initial answers to these questions.

Our experience-sampling study (Study 2), which sampled more than 4200 episodes across 10 days, uncovered some of the dynamics of (young) individuals’ daily social lives. Participants reported being with others in about 63% (and alone 37%) of the sampled episodes (which were throughout the day), and regardless of the social context, they were also in a setting of their choice in most (64%) of the episodes. These frequencies are consistent with findings reported in previous studies (e.g., Hudson et al., 2020; O'Connor & Rosenblood, 1996), and they imply that individuals (specifically, students) spend non-negligible periods—about a third of their time—in externally imposed social settings.

Do social interactions increase ESWB compared with periods of aloneness? The extant literature associates stable social relations with greater subjective well-being (Diener & Seligman, 2002), but findings are less conclusive for episodic social interactions (Uziel et al., 2020). The results of the present research coincide with the intricacy of the effect and provide directions toward understanding when and how episodic interactions affect well-being. First, being with others is associated with desirable effects if it reinforces one’s sense of agency, and it is detrimental in the absence of control. Supporting this account are our findings on the sense of control, which increased under chosen social settings, along with the increase in ESWB. These findings resonate early models about the effects of social presence in the social facilitation effect, which emphasized the role of (un)certainty in shaping the reaction to others’ presence (Guerin & Innes, 1982; Zajonc, 1965).

Another path for constructive (vs. destructive) episodic social interactions that emerges from the present findings concerns the sense of meaning. Social contacts were constructive when they were experienced as meaningful. Interestingly, low meaningful contacts (which in experiential sense are less impactful) were nonetheless associated with a relative reduction in ESWB, highlighting an often-neglected aspect in our daily social life. Furthermore, our findings imply that choosing (and perhaps initiating) social interactions is central in affecting ESWB, thus accounting for both—the reason why many people do not initiate such relations (because they generally expect to experience low ESWB in non-chosen settings), and why they may gain if acted to initiate (i.e., choose to be in such) interactions (Epley & Schroeder, 2014).

In popular and academic writings, episodes of aloneness are often depicted as reflecting reduced subjective well-being compared to social engagement (Larson, 1990; Srivastava, 2008). Our data lend partial support to these findings. Study 2 (but not Study 1) found periods of aloneness to be less conducive to well-being than 'with others' contexts, averaged across the different measures. Differences between these conditions were especially notable for sense of meaning. People felt that their actions were more meaningful 'with others' than alone (with the interaction term significant, but weaker than for other measures). This, though, does not necessarily imply that the alone setting was less desirable, as it could reflect the sense that having others observe your actions makes them more consequential (Baumeister, 1982).

Aloneness (by choice and not) emerged as a setting of relative stability, with participants experiencing their different alone conditions quite similarly. Therefore, solitude might not present immediate benefits to well-being, but it does appear to offer a more predictable experience, and if utilized effectively could be a source of personal growth (Lay et al., 2018; Long & Averill, 2003; Uziel, 2021). A worthy direction for future research would be to compare the immediate and sustained implications of periods of aloneness. Moreover, these findings imply that internal (i.e., non-contextual) factors play a significant role in shaping the effects of aloneness. Indeed, the literature has begun identifying relevant factors, such as personality traits (Uziel, 2016; Uziel et al., 2020), preferences and desires (Coplan et al., 2019; Leary et al., 2003), and developmental periods (Larson et al., 1985).

The most robust effect that emerged from the present two studies is in the intersection of being with others, aloneness, and choice. Choice was substantially more important 'with others' (vs. alone) in determining ESWB, sense of meaning, and control. This finding showed in controlled settings (Study 1) and real-life data (Study 2). This finding is in line with approaches stemming from laboratory research, which associate social presence with polarizing effects (Blascovich et al., 1999; Uziel, 2007, 2015), greater intensity and arousal (Wilt & Revelle, 2019; Zajonc, 1965), and self-presentational concerns (Baumeister, 1982). They are further in line with cognitive approaches suggesting that experiences are amplified in social presence (Boothby et al., 2014; Steinmetz et al., 2016). Our data indicate that for better or worse, experiences are more intense 'with others', and that choice of being with others is more consequential to well-being than the choice (vs. not) to be alone.

Last, this study highlights a relatively neglected aspect of research in social psychology, which often applies an experimental approach to the study of social interactions, and consequently non-chosen social settings. The findings inform about the role that chosen social settings play in real-life dynamics, showing that individuals often manage to navigate their social lives by their choice. It is worthwhile to consider this aspect with greater attention in future research.

## Limitations and Future Directions

The present research is not free from limitations. First, although sample composition varied between the two studies (e.g., by age and native language), participants were nonetheless from Western cultures (UK and Israel), and Study 2 participants were (mainly Female) college students. Perceptions and experiences of aloneness and of being with others may differ by culture and over the lifespan. Marital status, family composition, and work status could affect not only the likelihood of being with others (or alone) by choice (or not) but also one’s experience in these conditions. Future research could extend the present findings beyond the sampled populations and systematically consider the role of different life conditions.^3^

Second, the present studies were focused on transient situational variables, yet individual differences in personality may also affect the experiences in these settings. For example, being alone is experienced differently by individuals varying in neuroticism (Uziel et al., 2020) or in affinity for aloneness (Coplan et al., 2019). Seeking others' company is often affected by extraversion (Wilt & Revelle, 2019) and a range of additional personality traits (e.g., Uziel, 2015). Furthermore, locus of control and self-deception may moderate people's experience of situations as chosen or not.

A third issue concerns the scope of the experiences sampled. Our conclusions are bounded by sampling daily activities in the lives of normative populations. Questions of choice (or lack thereof) and solitude take different forms under extreme conditions, and this warrants separate investigations. Moreover, choice was considered in our study a (subjectively judged) dichotomy. It could be argued that situations are often a mix of choice and constraints. Future research could address this issue by considering different levels of experienced choice. In addition, although our Study 2 sampled a large number of episodes across and within days, it addressed experiences resulting from being in a given situation, but not dynamics resulting from these situations. Future research could address such dynamics by looking at situational contingencies (e.g., likelihood of being alone by choice after being with others), time spent in each situation, and variations in ESWB over extended periods. Additionally, we did not ask about the specific activities that participants were doing (nor about their level of engagement with other people in the ‘with others’ setting). Future research could extend the present findings by emphasizing the type of activities people pursue under these settings.

Relatedly, the present research was focused on self-related constructs. Future research could address implications associated with interpersonal variables (e.g., trust), and objective outcomes (e.g., physiological responses). An additional extension concerns intervention aiming to modify the perceptions of choice in (imposed) social contexts (e.g., while commuting) and their impact on ESWB.

## Conclusion

Social contexts and choice of being these contexts affect ESWB. The present findings reveal that their interaction matters, and that choice is associated with a stronger difference in ESWB when one is with other people than when one is alone. In terms of our momentary experiences, sensing that we are in the company of others by our choice is associated with the greatest boost to our well-being, sense of meaning, and control, whereas our time alone embodies an experience of relative stability.

## Footnotes

1 Study 2 was conducted in Hebrew. Materials were translated to English for ease of access.

2 Following a Reviewer’s suggestion, we also tested a 2-by-2-by-2 design, which included the physical setting (i.e., choice*social context*physical setting). Results revealed a non-significant 3-way interaction, *F*(1, 84) = 0.09, *p* = .754, η^2^_p_ = .001. The 2-way interaction between choice and social context was significant, and therefore not affected by the specific physical settings, *F*(1, 84) = 8.56, *p* = .004, η^2^_p_ = .092.

3 Another external factor that may affect people’s perception of their social experiences is the recent COVID-19 pandemic. We collected the data for Study 2 in 2019 (before the outbreak), and for Study 1 in 2021. The similarity in the main finding implies that the pandemic did not make a significant difference in affecting the specific dynamics we studied, but we hesitate to make strong claims at this point in time.
